# Dimensions of Interactive Pervasive Game Design: Systematic Review

**DOI:** 10.2196/42878

**Published:** 2023-08-22

**Authors:** Liu Kai, Wee Hoe Tan, Erni Marlina Saari

**Affiliations:** 1 School of Art and Design Henan University of Engineering Zhengzhou China; 2 Faculty of Art, Sustainability and Creative Industry Sultan Idris Education University Tanjong Malim Malaysia; 3 De Institute of Creative Arts and Design UCSI University Kuala Lumpur Malaysia; 4 Serious Games Association Singapore Singapore; 5 Faculty of Computing and Meta-Technology Sultan Idris Education University Tanjong Malim Malaysia

**Keywords:** interactive, pervasive game, systematic review, design, mobile phone

## Abstract

**Background:**

As the gaming industry grows around the world, playing pervasive games is becoming an important mode of entertainment. A pervasive game is one in which the game experience extends into the actual world or where the fictive world of the game merges with the physical world. How pervasive games can adapt to the ever-changing nature of technology and design in current society requires a comprehensive review.

**Objective:**

In this systematic review, we aimed to measure and analyze 4 dimensions of pervasive games through development, technology, experience, and evaluation. Moreover, we also aimed to discover and interpret their relationship with game, interaction, experience, and service design.

**Methods:**

We first chose 3 well-known databases, Web of Science, Scopus, and EBSCO, and searched from 2013 to April 2022. A strictly thorough Boolean search for research keywords such as “pervasive game,” “design,” and “interactive” resulted in 394 relevant articles. These articles were identified, screened, and checked for eligibility to find valid and useful articles, which were then categorized and analyzed using the PRISMA (Preferred Reporting Items for Systematic Reviews and Meta-Analyses) method.

**Results:**

The systematic selection was finally left with 40 valid and valuable articles. After categorization and analysis, all articles were classified according to 4 main themes, which were design and development (11/40, 28%), interaction and technology (15/40, 38%), users and experience (9/40, 23%), and evaluation and service (5/40, 13%). These 4 main areas can be subdivided into several smaller areas.

**Conclusions:**

In the 4 areas of game design, interaction design, experience design, and service design, many scholars have studied pervasive games and made contributions. Although the development and technology of pervasive games have evolved with the times, there is still a need to strengthen emerging design concepts within pervasive games.

## Introduction

### Background

During the global COVID-19 pandemic, people from all walks of life played video games to find joy, connection, and a sense of belonging. A game is a source of entertainment and comfort for millions of people in all countries, and it transcends age, race, gender, platform, and political party. In 2021, according to the Entertainment Software Association survey, 90% of gamers agreed that video games bring joy and strongly agreed that they can inspire (79%), provide mental stimulation (87%), and relieve stress (87%) [1]. Sensor Tower released its *Mobile Gaming 2022* report, which provides insight into how challenges affect different regions and game genres and which markets continue to grow in the face of these challenges. The biggest game opportunity is in China, as can be interpreted from the data. China currently leads the mobile game industry in terms of revenue and downloads and is expected to maintain its dominant position in the coming years [2]. Multiplayer online battle arena has proven to be one of the most challenging but rewarding game genres in the mobile game market, bringing a series of huge successes and painful failures. The latest high-profile multiplayer online battle arena to be released on cell phones is *Pokémon Unite*, developed by Pokémon Company in partnership with Tencent’s TiMi Studio. *Pokémon GO* was downloaded more than 15 million times in the first 2 days of its release, making it the biggest release ever. By the end of the first week, the game had reached approximately 30 million downloads in 7 days [3].

Pervasive games are a very important subfield among the many game classifications. As early as 2009, Montola et al [4] wrote in their book that pervasive games were defined as having *one or more salient features that expand the contractual magic circle of play spatially, temporally, or socially.* Initially, pervasive games were relatively simple to play. Game players with other players and nonplayer characters communicate via phone, email, or MSN Messenger [4]. However, now, pervasive games are a whole new game style that extends game experiences into the actual world, weaving into the fabric of players’ real-life settings [5]. According to some researchers, a pervasive game is when the player’s experience is extended to the actual world, achievable because of the device’s sensors [6]. These games are a relatively new type of entertainment that takes the game experience out of the device and into the actual environment, merging virtual and physical realities [7].

Pervasive games use mobile devices’ data collection, geotagging, e-commerce, internet cookies, and social media capabilities [8]. Pervasive games rose to popularity with the release of Niantic’s *Pokémon GO* in 2016. Two games now dominate the pervasive games market: *Pokémon GO* and *Ingress* [9]. Ingress has a rather simple core loop and low polish, but its dynamics seem to be tailored to enforce team socializing and collaboration [10]. After the successor to *Ingress*, *Pokémon GO* offers a more complicated primary gameplay loop. The mechanics of *Pokémon GO*’s main gameplay loop are more intertwined than those of *Ingress*; nonetheless, the game focuses significantly more on the individual’s experiences in the Pokémon world and far less on social connections and collaboration [11].

### Objectives

On the basis of the abovementioned background retrospect, it can be seen that further research on pervasive games is necessary. As design continues to evolve, many new concepts are being added. There is a need to add more current design factors in the game design industry. For example, interaction design, experience design, service design, and branding design are popular design concepts that constantly influence game design. In addition, the existing systematic review articles fail to provide detailed information on the review process. This includes keyword identification, article screening, and article eligibility. Moreover, because of this situation, prospective researchers could not reconstruct the survey, authorize interpretation, or assess the breadth of the data. Furthermore, this study is important because it provides researchers with an understanding of peer literature reviews and helps researchers better understand pervasive game design issues that may require academic attention. The current systematic analysis was conducted to answer the primary research question: Does the pervasive game embody other conceptions of thinking in the design field? The survey’s primary focus was on perceptions of pervasive game design, including perceptions of design thinking in interaction design, experience design, service design, and branding design.

The *Methods* section describes the procedure used to answer the research topic of this study. The *Results* section conducts a systematic review and synthesis of the scientific literature to identify, select, and analyze the research needed on pervasive game design within design thinking situations. Finally, the *Discussion* section discusses the steps that must be followed, emphasizing the importance of aspiring academics understanding the issues.

## Methods

### Overview

**Figure 1:**
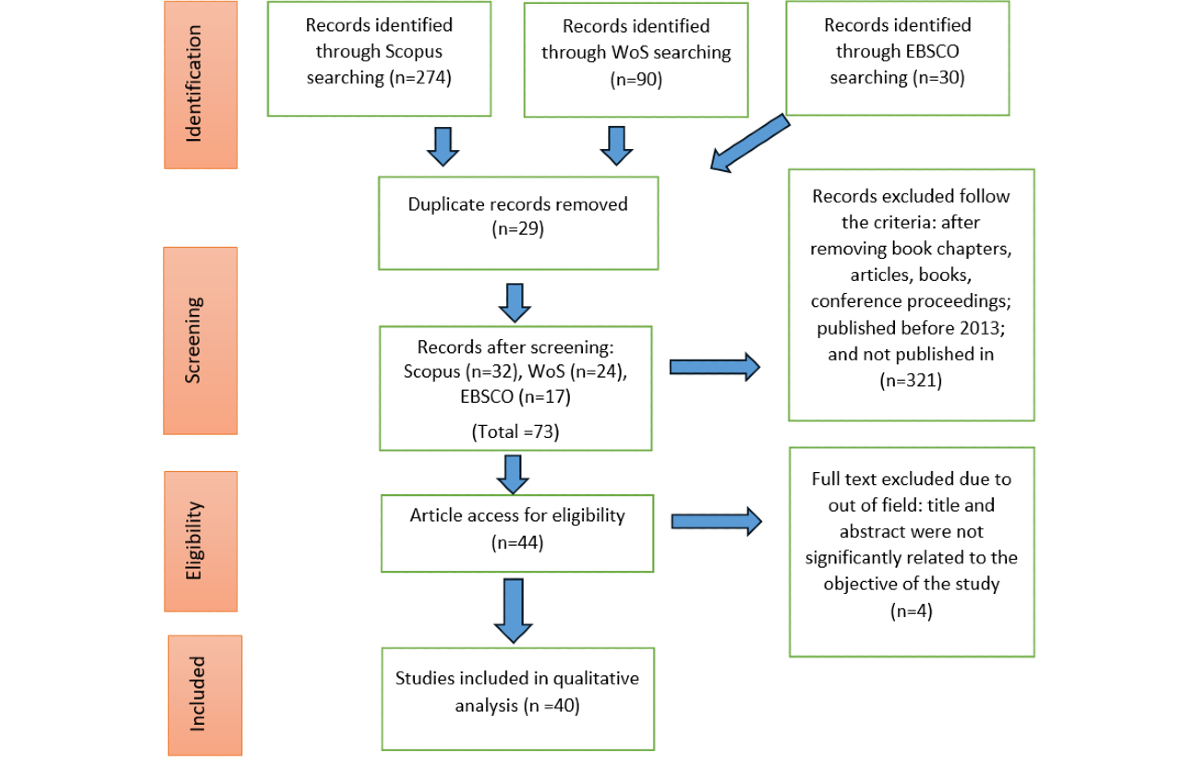
Flow diagram of the proposed search study. WoS: Web of Science.

Several studies related to system evaluation have been conducted globally. However, only a few studies have focused on the design thinking connotations of pervasive games in the context of design thinking [4-8]. This section describes the need for a systematic analysis of the design thinking connotations of pervasive games. In contrast, the following section presents the methodology used to find the research answers formulated by this study. This literature analysis is divided into four sections: (1) design and development, (2) interaction and technology, (3) user and experience, and (4) evaluation and service. Furthermore, this section systematically reviews and synthesizes the scientific literature to distinguish, screen, and analyze important pervasive game research. Finally, reflecting on potential scholars suggests what actions should be taken to address the issues raised. The prerecording systematic reviews and meta-analysis (PRISMA [Preferred Reporting Items for Systematic Reviews and Meta-Analyses]) approach was used in this analysis, a documented standard for conducting a systematic literature review. In general, publication rules are required to aid writers in assessing and reviewing the quality and rigor of a review with relevant and necessary details. PRISMA also emphasizes the randomized study evaluation survey, an important feature in systematic analysis reports for various study types [12] (Figure 1).

In terms of instruments, the resilient character of 3 electronic databases, Scopus, Web of Science, and EBSCO, was used to evaluate this research methodology. This review’s electronic database search included articles relevant to education, information technology, and social science. However, similar to Scopus, no database is perfect and detailed [13]. They mainly cover various fields, such as business and industry, economics, information technology, humanities, social sciences, communication and dissemination, education, arts, literature, medicine, and general science. This section also provides an overview of the 4 major subsections: identification, screening, eligibility, and data abstraction.

### Identification

The systematic review process can be divided into 3 main phases, each of which is responsible for selecting several appropriate papers for this report. The first step is the identification of keywords, followed by a search for related and analogous terms using resources such as the thesaurus, dictionaries, encyclopedias, and earlier research. Consequently, after determining all pertinent keywords, search strings for the databases Scopus, Web of Science, and EBSCO (Textbox 1) were formulated. This review successfully retrieved 394 papers from 3 databases during the first step of conducting a systematic review.

**Search strings.**

**Scopus**

- TITLE-ABS-KEY (”pervasive gam*” AND design* AND (interaction OR interactive)) AND (LIMIT-TO (PUBYEAR, 2022) OR LIMIT-TO (PUBYEAR, 2021) OR LIMIT-TO (PUBYEAR, 2020) OR LIMIT-TO (PUBYEAR, 2019) OR LIMIT-TO (PUBYEAR, 2018) OR LIMIT-TO (PUBYEAR, 2017) OR LIMIT-TO (PUBYEAR, 2016) OR LIMIT-TO (PUBYEAR, 2015) OR LIMIT-TO (PUBYEAR, 2014) OR LIMIT-TO (PUBYEAR, 2013)) AND (LIMIT-TO ( PUBSTAGE, ”final”)) AND (LIMIT-TO (DOCTYPE, ”ar”)) AND (LIMIT-TO (SRCTYPE, ”j”))

**Web of Science**

- ALL= (“pervasive gam*” AND design* AND (interaction OR interactive)) and Articles (Document Types) and 2022 or 2021 or 2020 or 2019 or 2018 or 2017 or 2016 or 2015 or 2014 or 2013 (Publication Years)

**EBSCO**

- ALL= (“pervasive gam*” AND design* AND (interaction OR interactive)) and Articles (Document Types) and 2022 or 2021 or 2020 or 2019 or 2018 or 2017 or 2016 or 2015 or 2014 or 2013 (Publication Years)

### Screening

During the preliminary screening, duplicate papers were eliminated from consideration. In the first phase, we decided not to include 29 articles. In the second phase, we used several inclusion and exclusion criteria developed to screen 73 articles. The first criterion was the literature, which consisted of research articles, as this is the most important source of useful information. Publications in the form of systematic reviews, reviews, meta-analyses, meta-syntheses, book series, books, chapters, and conference proceedings were excluded in the current investigation. In addition, the review focused solely on papers written in English. It is essential to remember that the plan was to select publications across 10 years, from 2013 to 2022. Thus, 321 publications were disregarded because they did not meet certain criteria.

### Eligibility

A total of 44 articles were prepared for the third step, which is referred to as eligibility. At this stage, the titles of all the articles and the primary content they contained were subjected to a comprehensive review to verify that the inclusion requirements had been reviewed and that the articles were suitable additions to this study following the research goals. Consequently, 4 reports were excluded because they did not consist of pure scientific articles based on empirical evidence. Finally, 40 articles were included in the final analysis (Textbox 2).

**Selection criteria.**

**Inclusion criteria**

- Language: English
- Timeline: 2013-2022
- Literature type: journal (only research articles)

**Exclusion criteria**

- Language: non-English
- Timeline: <2013
- Literature type: journal (book chapter and conference proceeding)

### Data Abstraction and Analysis

An integrative analysis, which is one of the examination techniques used to analyze and synthesize different research designs, was conducted in this study. This technique was used to integrate qualitative, quantitative, and mixed methods. The primary focus of the specialists’ research was on the formulation of pertinent topics and subtopics. The data collection phase was the initial step in developing the theme. We thoroughly combed through a collection of 40 papers, searching for statements or information that responded to questions raised by this study. The second step involved the expert and us analyzing the situation regarding pervasive games within design thinking, determining meaningful groups to form. The 4 primary themes that emerged from the design and development, interaction and technology, user and experience, and evaluation and service need to be overcome. We reviewed every developed theme from this point onward, including any themes, concepts, or ideas that have any connection. We established themes based on the findings of the study within the framework of this research. In this instance, a log was kept during the data analysis to document any analyses, opinions, puzzles, or other ideas pertinent to data interpretation.

We also compared the findings to resolve any inconsistencies that may have occurred while developing the theme. It is important to note that we discussed with academics any inconsistencies that may have occurred within the themes and tried to resolve the inconsistencies. In conclusion, the developed themes underwent minor adjustments to ensure coherence. Two specialists carried out the examinations: one specialized in the games industry and the other specialized in the game academic areas. This was performed to ensure that the problems were valid. By establishing domain validity, the expert review phase helped ensure that each subtheme was clear, important, and adequate. Adjustments were made at our discretion based on comments and feedback from industry professionals.

## Results

### Overview

By extending the game experience into the physical domain, pervasive games represent a new era of entertainment and interaction. Pervasive game design is one of the many issues that must be reconsidered. As the development of pervasive games becomes more and more successful, the need to incorporate new design concepts becomes increasingly urgent. There were 40 articles extracted and analyzed based on a searching technique. All articles were classified according to 4 main themes: design and development (11/40, 28%), interaction and technology (15/40, 38%), users and experience (9/40, 23%), and evaluation and service (5/40, 13%; Table 1).

**Table 1:** Detailed presentation of results based on the research criterion.

Study, year	Journal	Title	Available in the database?			Remarks
			Scopus	Web of Science	EBSCO	
Arango-López et al [14], 2021	*Universal Access in the Information Society*	GeoPGD: methodology for the design and development of geolocated pervasive games	Yes	Yes	No	Design and development
Arango-López et al [15], 2021	*Universal Access in the Information Society*	Using geolympus to create a pervasive game experience in the higher education context	No	Yes	No	Design and development
Costa et al [16], 2021	*Multimodal Technologies and Interaction*	An Interactive Information System that supports an augmented reality game in the context of game-based learning	Yes	Yes	No	Interaction and technology
Oliva-Maza et al [17], 2021	*IEEE Revista Iberoamericana de Tecnologias del Aprendizaje*	Mystery of the runaway letrabytes: inclusive assessment of phonological awareness with tangible gamification	No	Yes	No	Evaluation and service
Santos et al [18], 2021	*JMIR serious games*	Promoting physical activity in Japanese older adults using a social pervasive game: randomized controlled trial	Yes	Yes	No	Users and experience
Alavesa et al [19], 2020	*Multimedia Tools & Applications*	Embedding virtual environments into the physical world: memorability and co-presence in the context of pervasive location-based games	No	Yes	Yes	Interaction and technology
Bellotti et al [20], 2019	*IEEE Transactions on Games*	REAL: reality-enhanced applied games	Yes	No	No	Interaction and technology
Costa et al [21], 2020	*Information*	Design of a mobile augmented reality platform with game-based learning purposes	Yes	Yes	No	Interaction and technology
Bonillo et al [22], 2019	*Multimedia Tools & Applications*	Developing pervasive games in interactive spaces: the JUGUEMOS toolkit	Yes	Yes	Yes	Design and development
Eckstein et al [23], 2019	*Entertainment Computing*	Smart substitutional reality: integrating the smart home into virtual reality	Yes	Yes	No	Design and development
Leone [24], 2019	*IxD&A (Interaction Design and Architecture(s))*	U’Game—a toolkit for urban gaming	No	Yes	No	Interaction and technology
Santos et al [25], 2019	*JMIR serious games*	Effects of social interaction mechanics in pervasive games on the physical activity levels of older adults: Quasi-Experimental Study	Yes	Yes	No	Users and experience
Santos et al [26], 2019	*Journal of Rehabilitation and Assistive Technologies Engineering*	Pervasive game design to evaluate social interaction effects on levels of physical activity among older adults	No	Yes	No	Users and experience
Guo et al [27], 2018	*Information*	Ontology-based domain analysis for model driven pervasive game development	Yes	No	No	Design and development
Klaassen et al [28], 2018	*Sensors (Basel, Switzerland)*	Design and evaluation of a pervasive coaching and gamification platform for young diabetes patients	No	No	Yes	Evaluation and service
Valente et al [29], 2018	*Personal & Ubiquitous Computing*	A method to assess pervasive qualities in mobile games	Yes	No	Yes	Evaluation and service
Kasapakis and Gavalas [30], 2017	*Multimedia Tools and Applications*	Occlusion handling in outdoors augmented reality games	Yes	No	No	Interaction and technology
Maia et al [31], 2017	*Entertainment Computing*	LAGARTO: A LocAtion based Games AuthoRing TOol enhanced with augmented reality features	Yes	Yes	No	Design and development
Valente et al [7], 2017	*Requirements Engineering*	Mapping quality requirements for pervasive mobile games	Yes	No	No	Evaluation and service
Jong [32], 2016	*British Journal of Educational Technology*	Teachers’ concerns about adopting constructivist online game-based learning in formal curriculum teaching: the VISOLE^a^ experience	No	No	Yes	Users and experience
Mora et al [33], 2016	*Journal of Ambient Intelligence & Smart Environments*	From interactive surfaces to interactive game pieces in hybrid board games	No	Yes	Yes	Interaction and technology
Sekhavat [34], 2016	*International Journal of Computer Games Technology*	KioskAR: an augmented reality game as a new business model to present artworks	No	No	Yes	Interaction and technology
Guo et al [35], 2015	*International Journal of Multimedia and Ubiquitous Engineering*	RealCoins: A case study of enhanced model driven development for pervasive games	Yes	No	No	Design and development
Kasapakis and Gavalas D [36], 2015	*Journal of Network & Computer Applications*	Pervasive gaming: status, trends and design principles	No	No	Yes	Design and development
Kasapakis et al [5], 2015	*Personal and Ubiquitous Computing*	Pervasive games field trials: recruitment of eligible participants through preliminary game phases	Yes	No	No	Evaluation and service
Lemcke et al [37], 2015	*Personal and Ubiquitous Computing*	RouteMe: a multilevel pervasive game on mobile ad hoc routing	Yes	No	No	Interaction and technology
Lv et al [38], 2015	*Personal & Ubiquitous Computing*	Touchless interactive augmented reality game on vision-based wearable device	Yes	No	No	Interaction and technology
Pløhn et al [39], 2015	*Electronic Journal of e-Learning*	Dynamic pervasive storytelling in long lasting learning games	Yes	Yes	No	Design and development
El-Nasr et al [40], 2015	*Entertainment Computing*	Data-Driven Retrospective Interviewing (DDRI): A proposed methodology for formative evaluation of pervasive games	Yes	No	No	Evaluation and service
Sra and Schmandt [41], 2015	*Personal & Ubiquitous Computing*	Expanding social mobile games beyond the device screen	Yes	Yes	No	Interaction and technology
Evans et al [42], 2014	*Personal & Ubiquitous Computing*	The Malthusian Paradox: performance in an alternate reality game	Yes	Yes	Yes	Interaction and technology
Maggiorini et al [43], 2014	*Multimedia Systems*	Opportunistic mobile games using public transportation systems: a deployability study	Yes	No	Yes	Interaction and technology
Schmitz et al [44], 2014	*Pervasive and Mobile Computing*	The impact of coupled games on the learning experience of learners at risk: an empirical study	Yes	No	No	Users and experience
Chamberlain et al [45], 2013	*Entertainment Computing*	Them and Us: an indoor pervasive gaming experience	Yes	Yes	No	Users and experience
Chen et al [46], 2013	*International Journal of Human-Computer Interaction*	Your way your missions: A location-aware pervasive game exploiting the routes of players	Yes	Yes	Yes	Interaction and technology
Coenen et al [47], 2013	*Journal on Computing and Cultural Heritage*	MuseUs: case study of a pervasive cultural heritage serious game	Yes	No	No	Interaction and technology
Kristiansen [48], 2013	*International Journal of Mobile Human Computer Interaction*	Design games for in situ design	Yes	Yes	No	Design and development
Neustaedter et al [49], 2013	*Personal & Ubiquitous Computing*	Creating scalable location-based games: lessons from Geocaching	Yes	Yes	Yes	Design and development
Papagiannakis et al [50], 2013	*Journal of Universal Computer Science*	A multimodal ambient intelligence environment for playful learning	Yes	Yes	No	Users and experience
Soute et al [51], 2013	*Entertainment Computing*	Evaluating player experience for children’s outdoor pervasive games	Yes	No	No	Users and experience

^a^VISOLE: Virtual Interactive Student-Oriented Learning Environment.

### Design and Development

#### Development Mode

Pervasive games are a completely new game form that extends game experiences into the physical world by incorporating information and communication technologies into the fabric of players’ real-world environments. This emerging game mindset makes it difficult for developers to explore technologies and methods for providing a high-quality interactive experience for users and designing novel and compelling forms of content [36].

Model-driven development and domain-specific modeling have succeeded in many open or in-house information system scenarios. However, its application to games is rare and immature. Guo et al [35] illustrated solutions by describing a pervasive game case’s technical and procedural development, incorporating model-driven development into the game development process. Furthermore, Guo et al [27] proposed a lightweight domain analysis that can be incorporated into the pervasive game development process. The domain analysis process is based on a game ontology that provides game design and domain analysis. They introduced the ontology, demonstrated its application in the domain analysis procedure, and evaluated and discussed the quality of the ontology using a user acceptance survey. The test results indicated that most potential users found the ontology useful and simple to use.

#### Development Tool

When developing pervasive games, programmers must deal with high turnover, cheating, and heterogeneity of devices and platforms. LAGARTO is an authoring tool for constructing location-based mobile games with augmented reality (AR) capabilities. These games are a subclass of pervasive games in which the game’s progression depends on the location of the players. Maia et al [31] gleaned requirements for designing the LAGARTO software architecture from a literature review and focus group meetings. The tool consists of a web-based application for creating and managing games and a mobile app for tracking players. Their primary objective is to develop software that enables nonprogrammers to design, construct, and run location-based mobile games. The development of pervasive games is slowing down because of the multiple challenges that these games present to their creators, such as the vast array of interaction paradigms and the complexity of developing applications where so many innovative technologies converge. Bonillo et al [22] introduced JUGUEMOS, a toolkit designed to assist developers in creating pervasive games for interactive spaces. The toolkit addresses 3 difficulties that arise during the development of pervasive games: integrating heterogeneous devices, managing multiple displays, and facilitating game coding. They conducted case studies exploring the toolkit’s expressivity, its ability to support collaborative multidisciplinary experiences, and its potential to support interactive experiences outside our interactive space. With the addition of pervasive or narrative components, however, the complexity of construction and support increases, necessitating a tool to manage the information appropriately and dynamically. Space and the pervasiveness of social interaction collaborate to achieve objectives by exchanging data between multiple pervasive games to improve the game experience. Arango-López et al [15] described a platform (*Geolympus*) that enables the creation of game experiences based on the player’s location.

#### Development Direction

In recent years, games’ design and development incorporating web-based elements into real environments have expanded. Such games include pervasive games, which seek to enrich the game world by combining these 2 realities to immerse the player more deeply in the story. The implementation of pervasive games has increased player engagement and motivation. New technologies have enabled advancements in the design and development of video games. Pervasive games have not been an exception, and combining traditional game elements with real-world elements and scenarios has made it possible to enhance motivation and user experience. However, previous research has demonstrated the need for a procedure to guide the design and development of pervasive games. In light of this, Arango-López et al [14] developed GeoPGD, a methodology that integrates the design of geolocated narrative as the core of the game experience. This methodology guides designers and developers through the various stages of creating pervasive games and provides tools for defining narrative elements, locations, and user-pervasive game interactions. Furthermore, Pløhn et al [39] described the Dynamic Pervasive Storytelling (DPS) approach and the design of the pervasive game *Nuclear Mayhem*, which was created to support a course in web game development at Nord-Trndelag University College in Norway. *Nuclear Mayhem* runs alongside the course for 9 weeks and requires specific elements to become part of the players’ everyday lives, reminding them of the game they are playing. The game DPS as a model aims to increase the universality of the game and support the player’s ongoing level of awareness in the game by incorporating real-world events. DPS uses real-world events as a building block to create the entire game story before the game begins by incorporating current events into its design and increasing the universality of the experience and in-game awareness as the game unfolds. This paper concludes that DPS is a promising approach to developing game narratives because it increases the universality of the game and promotes in-game awareness.

Mobile culture has given rise to many context-based products such as location- and hashtag-based apps. This poses new challenges for designers. Design methodologies that recognize context and allow it to influence design concepts are required. Kristiansen [48] focused on a design problem where live design practices can enhance the early design process: designing a pervasive game. Pervasive games are computer games that use cities as playgrounds and cell phones with GPS capabilities. Some contextual design approaches already exist, but the authors propose an approach that requires designers to conceptualize and implement ideas in the field, that is, at the game’s location. The challenge is to develop a creative approach that includes on-site design work and generates game concepts for pervasive games. The proposed design approach, called *site storming*, was based on games involving individual scenario exploration of the site and various types of game cards, followed by a collective evaluation of the ideas generated. A series of evaluations showed that designers enjoyed using this method, stimulated idea generation, and live design influenced their design concepts.

The pervasive game aims to take computer games out of the PC and into the “real world” of cities, streets, and parks. This real-world physicality makes for an enjoyable player experience but presents a unique challenge for designing and coordinating such games. Neustaedter et al [49] explored how location-based games can be designed to overcome this scalability challenge—using Geocaching to determine how it has maintained user engagement and growth over the past decade. The findings show that Geocaching benefits from direct player creation of simple and complex game content. Geocaching also simplifies game choreography by allowing players to monitor game content, other players, and even nonplayers. Recently, developments in virtual reality technology have allowed users to consume immersive content from the comfort of their living rooms. Alternate reality promises to enhance this experience by incorporating physical environments into the simulation. Smart substitute reality (SSR) is a novel approach that combines the passive haptics of substitute reality with the active capabilities of a smart home environment. SSR is intended to serve as the basis for serious games. Eckstein et al [23] discussed the concept of SSR while implementing a prototype in our Smart Lab. They created multiple web-based environments with varying degrees of mismatch to the real world and added selected objects to induce additional tactile and thermal stimuli to enhance immersion.

### Interaction and Technology

#### Outdoor Location

With the rise of pervasive computing, location-aware pervasive games are increasing rapidly. Chen et al [46] presented the design and implementation of *Your Way Your Missions*. This location-aware pervasive game uses the player’s route to combat the inefficiencies of location- and radius-based information adaptation. *Your Way Your Missions* provides the player with a Google Maps–based tool to predefine the route and relies on self-reporting to obtain the player’s planned route. Such a design returns the task to the player based on the task’s location attributes and the player’s intended route. The results show that the route predefinition and self-reporting approach is an effective way to obtain the player’s planned route. This adaptation of information based on the player’s planned route can help them locate the task at any time and location. Leone [24] also described this location-based outdoor interaction in his article. He described this small community’s view of the connection between the real world and the video game world using physical and digital support. The paper highlights 3 games produced through participatory processes and argues for the role of urban and popular games as tools to promote citizenship. Other scholars have also suggested that using augmented or web-based field trips and pervasive educational games can potentially transfer learning content to the real world outside the classroom. Lemcke et al [37] presented the pervasive educational game *RouteMe*, which contextualizes the rather abstract topic of routing in ad hoc networks. The game was designed for college-level courses as a motivational supplement to these courses to enhance the learning experience. Students take on the role of routing nodes or applications with routing requirements. In 3 successive levels of difficulty, they are introduced to game concepts, learn basic routing mechanics, and become aware of the general limitations and capabilities of routing nodes.

With the increasing popularity of personal communication devices, the demand for location- and environment-based mobile services is growing exponentially. The mobile game as a service is no exception. Unfortunately, unlike other services, location- and context-based games strictly require near-field communication to create teams and arenas with nearby players. As currently used technologies have scalability (Bluetooth) or energy (Wi-Fi) limitations, opportunistic networks are considered a viable solution to engage large numbers of players in a larger area. However, it remains unclear how the increased latency and probabilistic message forwarding introduced by opportunistic networks will affect the player experience. Maggiorini et al [43] addressed these issues by simulating the deployment of a connection-based game on opportunistic networks provided by public transportation systems in 3 cities: Milan (Italy), Edmonton (Alberta, Canada), and Chicago (Illinois, United States). Moreover, they studied an opportunistic cooperative version of a well-known independent game for playability and scalability reasons. The emphasis on this particular game promotes the use of public transportation systems. Emerging pervasive games use sensors, graphics, and web technologies to provide immersive gameplay interactions integrated with the real world. Existing pervasive games typically rely on the device screen to provide game-related information and ignore opportunities to include new types of contextual interactions such as jumping, punching gestures, or even voice as game input. Sra and Schmandt [41] presented the design of *Spellbound*, a team-based physical mobile game, to help us understand how to design pervasive games that foster a spirit of togetherness. Sra and Schmandt [41] also briefly discuss how solidarity and playfulness transform physical movements into an ideal activity. *Spellbound* is an outdoor, team-based physical game. It takes advantage of the aforementioned opportunities to combine real-world movements such as jumping and spinning with the web-based world. It also replaces touch-based input with voice interaction. It uses custom hardware to provide tactile feedback, embodying the true spirit of social play that characterizes traditional children’s games. They believe that *Spellbound* is a form of digital outdoor play anchoring enjoyment in physical movement, social interaction, and tangible feedback.

#### Industry Application

Pervasive games have also contributed in terms of industry domains. Coenen et al [47] presented a case study of *MuseUs*, a popular pervasive game for museums that operates as a mobile phone app. During the museum visit, players are encouraged to create their exhibits and are guided through the process by application. The aim was to provide a learning effect during a museum exhibit visit. The core of the *MuseUs* experience is that it does not require a predetermined path through the museum and does not interfere with the exhibit itself. In addition, the application encourages visitors to see elements of cultural heritage in a new light, enabling a personal narrative while building a personal exhibition. Moreover, Sekhavat [34] described the architecture of *KioskAR*, a pervasive AR game. This game introduces a new business model that enables players to use AR to display their artworks in a web-based kiosk while enjoying the game. In addition to the competition, this game requires social interaction between players to earn more points. A user study evaluated the presence and usability of the application. The experiment showed that *KioskAR* could achieve a high level of usability and presence.

#### Interaction Mode

Creating pervasive games using emerging interactive technologies is becoming increasingly popular. Lv et al [38] designed and evaluated a touchless motion interaction technique for developing interactive and AR games on vision-based wearable devices. Users interact with AR games by making dynamic hand or foot gestures in front of the camera, which triggers interactive events with objects in the scene. As evidence, 3 elementary AR games with 11 dynamic gestures were created using the proposed touchless interaction technique. Finally, they presented a comparative evaluation to demonstrate the social acceptability and usability of the touchless approach running on a hybrid wearable frame or Google Glass and assess workload, user sentiment, and satisfaction. Recently, developments in interactive surfaces and tangible user interfaces offer new opportunities for pervasive games that combine traditional board games’ social affordances with the interactivity of video games. Mora et al [33] proposed a strategy centered on tokens, constraints, spatial representations, and interactive events in this research area. Rather than using interactive surfaces as the primary interaction medium, this approach relies on physically manipulating interactive, computer-enhanced game components on standard surfaces. Furthermore, AR in mobile game development, where computer-generated graphics are used to enhance the player’s view of the physical world, is becoming increasingly common.

#### AR Overview

In AR applications, it is common for objects to be partially or completely obscured by physical objects; if not handled properly, the visualization of obscured objects can often mislead the user’s perception. Kasapakis and Gavalas [30] presented 3 alternative geolocative raycasting techniques designed to help outdoor pervasive game developers solve the occlusion problem by integrating real-time building recognition to generate a realistic field of view for the player. The geographic raycasting approach has been implemented in the location-based pervasive game *Eliminate Order*, which uses publicly available free building data to calculate the player’s field of view in real time. The proposed algorithm applies to many sensor-based AR applications and can be ported to any real-world environment. A few years earlier, Evans et al [42] examined the design of the *Malthusian Paradox* and highlighted how it redefines the framework of performance in sometimes disturbing ways by blurring the traditional roles of performer and audience. Players engage with the game through various interactive channels, including performative group performances and one-on-one interactions with game characters in public settings, using low-end and high-tech physical and web-based artifacts such as customization and third-party websites. Players and game characters communicate in planned and unplanned ways via phone and social media. We reflect on the production and choreography of the game, including the dynamic nature of strong episodic narratives driven by professionally produced short films that attempt to respond to the player’s actions and the difficulties of designing for engagement across hybrid and time-dilated performance spaces.

Realistic 3D environments, such as existing city models, have the potential to serve as a bridge between the physical and the environments in pervasive games. Smooth attention shifts and transitions between these 2 realities are largely unexplored in the context of pervasive games. Alavesa et al [19] conducted 2 field experiments using a pervasive live-action role-playing game to investigate the effects of transitions between the web-based and physical environments on coexistence and memorability. Although there were few differences in copresence during play, they highlighted the subtleties in the social structure of the universal game. 3D environments are more memorable because of their spatial similarities to the physical world. We then identified 2 important factors to consider when integrating web-based environments into pervasive games: the structuring of social interactions and spatial realism. The emerging genre of pervasive games combines reality with computation. Bellotti et al [20] explored the concept of “reality-enhanced games” through a simple set of pervasive games. A reality-enhanced game is a model that links game mechanics to the outcomes or measurements of real-world activities. The prototype game was created as part of the TEAM Industrial Research Project, which aims to develop mobile apps for flexible and collaborative mobility. The proposed game is an example of various user interfaces that are believed to be useful for various important scenarios, goals, and user types, particularly for improving driving styles. This concept allows pervasive game developers to focus on their game logic while seamlessly leveraging a variety of in-field sensors, and it is generic.

AR is an emerging technology that superimposes objects onto the physical world. Recent computer and mobile technology advances have made AR increasingly accessible in education. Costa et al [21] described an educational mobile AR platform. The platform includes a mobile app consisting of a pervasive game designed to encourage learning about the universe. In addition, it includes a backend that enables teachers to present information about celestial bodies and create multiple-choice questions to assess students’ knowledge of the participants they are studying. The mobile app provides users with real-world physical movement and social interaction while playing the game; therefore, it falls within the paradigm of pervasive games. In addition to engaging students to play games, they believe that this platform can be implemented in both informal and formal learning environments. Mobile AR applications are gaining prominence in education, but there is a need to design educational games that are both appropriate and enjoyable. Costa et al [16] described an interactive information system to facilitate the implementation of a pervasive game for game-based learning. *PlanetarySystemGO* includes a location-based mobile AR game that facilitates learning about cosmic objects and planetary systems and a web application that interacts with mobile device apps. This resource can be used for web-based and face-to-face classes, which are useful in socially isolated situations, such as those caused by the COVID-19 pandemic. In addition, adding web applications with a backend to the information system allows for the inclusion of curriculum content based on the student’s grade level. Similarly, teachers are expected to use the information system to incorporate content that they feel is appropriate for their teaching grade level. Therefore, it is critical to provide them with the professional development they need to use this resource. Teachers found this resource helpful in motivating students to learn, recognizing that the web application facilitated the introduction of appropriate curriculum content and was useful in assessing student performance in games.

### Users and Experience

#### Children’s User Experience

There is a growing body of research on pervasive outdoor games, with most studies focusing on adult mobile phone players. Most published assessments of player experience in such games are based on anecdotal descriptions and postgame surveys. The latter approach is particularly difficult to implement when the game testers are children. Until now, observations of games have been ad hoc and based on unstructured observations, making it difficult to draw reliable conclusions from observations and compare games. The Outdoor Play Observation Scheme and GroupSorter are 2 methods developed specifically to assess the player experience of children’s outdoor play [51]. They discussed their application in 3 case studies and concluded that Outdoor Play Observation Scheme is useful for quantifying various play behaviors in outdoor play. GroupSorter adds qualitative data about play experiences. Moreover, the application of GroupSorter is not limited to game development; it can be used to collect user input in other contexts. In the same year, Papagiannakis et al [50] described the design, development, and evaluation of AmI Playfield, a technical framework for learning applications designed to create challenging learning conditions through play. An AmI Playfield is a guided ambient intelligence. It emphasizes using kinesthetic and collaborative technologies in a natural, play-based learning environment. It also incorporates performance measurement techniques. The *Apple Hunt* application was created to test and evaluate AmI Playfield, engaging child learners in arithmetic thinking through kinesthetic and collaborative play. Simultaneously, AmI technology observes behind the scenes. *Apple Hunt* has been assessed using a combination of methods suitable for young testers, and the Children’s Commission is presented as a promising assessment method for children. The results indicate the system’s high capacity to stimulate thinking and enjoyment, thanks to the learners’ whole-body kinesthetic play and teamwork.

Acquiring literacy skills requires developing phonological awareness, a metacognitive skill. This allows thinking about and manipulating language structures from an early age. Learning requires multisensory stimulation as well as curiosity and interest. Communication technologies play an important role in this, as learning-oriented use can stimulate interest and thinking in current digital natives. Oliva-Maza et al [17] described a pervasive game with tangible technology that can be used for interventions and diagnostic, formative, and summative assessments. Multiple case studies were conducted with 4 kindergarteners over 2 weeks to test the user experience. They were given a multimedia pervasive adventure game in which they had to use tangible technology to solve phonological awareness challenges to reach the final goal. Observations were recorded in an evaluation model for posttriangulation data analysis and subsequent qualitative analysis of pre- and postintervention data. The results showed improvements in phonological awareness and traces of gamified experiences. These encouraging results justify the application of these methodologies to a learner-centered education model.

#### Adults’ User Experience

The emergence of pervasive technologies has sparked an interest in designing and developing pervasive games. There are numerous designs of pervasive games for adult users. Chamberlain et al [45] presented *Them and Us*, a pervasive indoor game that uses dramatic processes to encourage social interaction within the context of the game. The *Them and Us* gameplay brings a group of people together in a space to interact with each other. At the same time, location-based technology reveals *us* the locational nature of the social interactions occurring in the space (who, where, and when). It allows *us* to score based on these interactions. *Them and Us* took a narrative-based approach in which a script instructed participants on how to play the game and how their social behaviors were incorporated into the game mechanics. This new interactive game-based artwork blends the player’s physical and web-based worlds to create a new and exciting experience for *them*. Designed specifically for adults, the pervasive game—*Them and Us*—emphasizes the use of audience and performer tracking as a means of encouraging interaction within the play space.

The pervasive game has also made a significant contribution to the education of adults. Providing pervasive game-based learning scenarios for at-risk students is effective and motivating. Schmitz et al [44] provided a detailed example of an educational environment in which a mobile game is combined with a PC browser game. They evaluated how this pairing helps the target group engage and learn. The study surveyed 19 people, ranging in age from 17 to 21 years, who participated in the game and explored it. The results of the 7-week game study suggest that pervasive games can increase learners’ interest in a topic while also supporting learning activities. Other studies have been conducted in the context of intense debate about how pervasive games can be used to provide new constructivist learning opportunities for students. Jong [32] developed a theoretical foundation for the Virtual Interactive Student-Oriented Learning Environment (VISOLE). It was a pedagogical framework for implementing constructive web-based gamified learning in teaching formal curricula. In addition to highlighting the pedagogical features of VISOLE, Jong [32] discussed the Stages of Concern model, which investigates 118 teachers’ concerns about this educational innovation in terms of 5 categorical concerns: evaluation, information, management, consequences, and refocusing. All participants had completed introductory VISOLE training before taking the Stages of Concern survey. He was also able to develop more precise interventions tailored to the actual needs of teachers to help him use this *diagnostic* knowledge to implement VISOLE in their schools. The findings also shed light on how to design, develop, or adapt pervasive games to aid learning and teaching in the classroom.

#### Older Adults’ User Experience

Promoting active lifestyles among older adults can have a significant positive impact on their quality of life. Pervasive games aim to create more fun and engaging experience by incorporating real-world elements into the game. The innovative mechanics of pervasive games, combining real and web-based worlds, can further engage and motivate older adults to achieve this goal. They can be a powerful strategy to encourage physical activity among older adults as they are integrated into players’ lives and naturally promote more casual play. Santos et al [26] designed and evaluated the feasibility of a pervasive game using social interaction as a case study to understand how game design elements can influence the physical activity levels of older adults. They created a mobile, location-based pervasive game and tested its feasibility as an experimental system among community-dwelling older volunteers in Kyoto, Japan. Participants found the theme and visual style of the game appropriate, and the rules and objectives of the game were easy to understand. The game was praised for its challenging and entertaining nature. Further research suggests that future iterations of the system will require special attention to the complexity of the controls and new ways to connect players when few people are playing or when they are too far apart.

In the same year, the goal of Santos et al [25] was to test the impact of changing a specific design element of a popular game for older adults, namely social interaction, on physical activity levels. Therefore, they experimented by comparing 2 variations of the same popular game over 4 weeks: the test group received social interaction, whereas the control group received no social interaction. In both versions, players had to walk to physical locations to collect cards, but the socially interactive version allowed people to cooperate in obtaining more cards. The impact on each group was assessed using the number of steps taken per week, and the number of places visited was used to measure game activity. A total of 32 people were recruited for the experiment (no social interaction=15; social interaction=17); 18 people persisted until the end (no social interaction=7; social interaction=11). The experiment found that the social interaction design element of the popularity game may have had some positive effects on promoting physical activity. Subsequently, in 2021, Santos et al [18] continued with the same study, comparing 2 variations of the same pervasive game over 4 weeks with the same purpose and type of experiment: the test group received social interaction whereas the control group did not. In this test, 20 participants were recruited for this experiment (no social interaction group, n=10; social interaction group, n=10), 18 of whom remained active until the end of the study (no social interaction group, n=9). After several tests of pervasive games with older adults, they concluded that the social interaction design element of pervasive games might positively affect physical activity. Although other factors may have influenced this effect, it has tremendous implications for the fitness and health of older adults.

### Evaluation and Service

#### Evaluation in the Area of Health

Recently, games have gained attention as a medium for changing health behaviors. However, current projects on healthy games face a major obstacle: the evaluation methods used to assess and formally evaluate such games do not adequately measure the acceptability and integration of the games into participants’ lives. These are important components of ensuring participants adhere to the games to induce behavior change. El-Nasr et al [40] described the data-driven retrospective interviewing method that we developed for formative evaluations. Used in a naturalistic setting, the data-driven retrospective interviewing examines how participants embrace the game and integrate it into their lives. The procedure consists of several steps. First, a game is designed to collect behavioral data, which are then analyzed to provide a basis for developing interview questions that retrospectively understand and reflect participants’ behavior. The contribution of this paper lies in its assessment methodology and application to *SpaPlay*, a universal health game [52]. As a result of this investigation, designers could rethink and improve their game design as they discovered that users were not using the game as initially envisioned. Such results would not have been possible with traditional evaluation techniques.

Self-monitoring, goal setting and coaching, education, and social support are daily care strategies for patients with chronic diseases. Various tools, such as mobile digital coaching systems linked to wearable sensors, pervasive games, and patient portals for personal health records, have been developed to assist patients with chronic conditions and their caregivers in achieving the ideal of self-management. Klaassen et al [28] described a platform that integrates these tools to help young patients self-manage their diabetes through educational games, monitoring, and motivational feedback. The platform’s design references health care, persuasive system design, and serious game design principles. A coach is a game guide that provides personalized feedback on the user’s daily care-related activities and helps them progress in the game world. User evaluations with patients under pediatric supervision suggest that using mobile technology in conjunction with web-based elements is feasible. However, assumptions about how users connect to the platform are not met in practice, resulting in a suboptimal user experience. They discuss the challenges and provide recommendations for advancing the integration of coaching and gamification platforms prevalent in medical practices.

#### Evaluation in Other Areas

Some scholars have explored a new approach to conducting user evaluation trials for pervasive games. In a case study, Kasapakis et al [5] examined the evaluation process of the pervasive role-playing game *Barbaros* consisting of preliminary and main implementation phases. The former is freely accessible to anyone and can be played at any time or place without the investigator’s team’s organizational and coordination investment. The latter defines 3 interdependent player roles that must cooperate in the scavenger hunt to achieve a common game goal. Players’ eligibility to participate in the main game phase is determined by their position in the previous phase. Using concepts from cultural theory, they designed the preparation phase as a dynamic environment from which potential evaluators would emerge. The main hypothesis being investigated is that implementing such a cost-effective preparation phase could recruit highly qualified participants for user trials of the pervasive game study prototype, thereby increasing the reliability and quality of evaluation results.

The requirement’s engineering community has not yet given enough attention to games. It becomes even more critical as we move toward newer forms of games, such as pervasive games. Pervasiveness (the characteristic that distinguishes traditional digital games) can mean being everywhere, pervading something, or propagating something through physical space. Each time a game is played, the boundaries of the game expand from the fictional world to the real world, indicating pervasiveness. Pervasive games, also known as alternate reality games, transmedia games, and cross-media games, are a new type of digital entertainment that has developed in various forms. The rapid development of this new type of digital product can be attributed to sensor technologies, networking capabilities, AR systems, computer vision technologies, the internet, and, in particular, mobile devices. Valente et al [7] defined it as pervasive games played on mobile devices. Mobile devices are currently the main drivers for the promise of pervasive games. Using various information sources, they compiled a list of interrelated characteristics critical to the success of these games. They presented a 2-level conceptual map of nonfunctional requirements based on these fundamentals to help achieve pervasiveness in pervasive mobile games.

Furthermore, Valente et al [29] proposed a new method for evaluating the quality of pervasiveness in pervasive games that can be adapted and applied to other pervasive applications. This approach produces a quality report consisting of a quality spreadsheet (containing metric values and comments) and a quality vector (representing the game quality profile in the form of a histogram). In addition, they can compare quality vectors based on similarity criteria. They applied the proposed approach to commercial and academic prototype games to elucidate their universality characteristics and identify ways to improve the overall quality of these games. In this sense, what distinguishes them from traditional digital games is the universality.

## Discussion

### Principal Findings

The analysis and results of the systematic literature review broadly divided these articles into 4 directions. These 4 directions are the direction of design and development, the direction of interaction and technology, the direction of the user and experience, and the direction of evaluation and service. These 4 directions appear to be independent of each other, but they are interdependent, which is consistent with the idea of this study. At this point, these directions can be correlated to the research objectives of this study one by one. Therefore, after abstracting and simplifying the themes of these literature analysis results, it is possible to sublimate them into corresponding design fields. Namely, the direction of design and development corresponds to the field of game design; the direction of interaction and technology corresponds to the field of interaction design; the direction of user and experience corresponds to the field of experience design; and the direction of evaluation and service corresponds to the field of service design. Therefore, these 4 design fields are the 4 research objectives to be analyzed in this study: game design, interaction design, experience design, and service design. They are also important indicators for analyzing the pervasive game from the perspective of different design thinking.

The game design aspect of pervasive games presents polymorphic research, which can be roughly divided into 3 categories: the development mode of game design, the development tool of game design, and the development direction of game design. Because pervasive games are a class of games, they naturally have a game design and development properties. As early as 2015, Kasapakis and Gavalas [36] introduced the historical development lineage of pervasive games, player acceptance factors, game development principles, and future development trends by analyzing 2 generations of 18 successful pervasive games. Subsequently, scholars have proposed different toolkits for developing pervasive games, enriching the toolbox of pervasive game design [15,22,31]. Moreover, the development direction of pervasive game design has never stopped research from 2013 to 2021. Inside these ideas are those that let players create game content directly [49], those that let designers find inspiration in the field [48], and those that let the game itself incorporate smart homes [23]. Furthermore, some research studies pervasive games from a storytelling and narrative perspective. This approach guides designers and developers through the different stages of game development, providing tools to define narrative elements, locations, and interactions between users and the game [15,39]. It is evident from looking at various aspects of pervasive game design, a popular focus of scholars’ research, showing a promising trend of multifaceted and multidimensional development.

The interaction design aspect of pervasive games has been the most studied by scholars, comprising 15 articles. It is only natural that this is the case, as pervasive games are a large category of games, and players must have a variety of interaction designs or interaction technologies in them when they play. First, pervasive games are location-based games, so there is a large body of scholarship on how they use interactive technology to interact outdoors [24,37,41,43,46]. Second, there were scholars in industrial applications to study how pervasive games can increase interactive communication of artworks and how to improve interactive communication of cultural heritage in museums [34,47]. Touchless interaction is then used in designing the interactive interface and physical user interface of pervasive games [33,38]. Finally, scholars have mostly studied pervasive games combined with AR [16,19-21,30,42]. Furthermore, it can be seen that the technological development factor in the interaction design of pervasive games is influenced. Owing to the limitations of mobile technology in the early years, the interaction design of pervasive games was most explored by SMS text messaging and Bluetooth [42]. By 2021, scholars were exploring ways to integrate AR into the interaction design of pervasive games [16]. Nevertheless, these studies continue to expand the boundaries of interaction design for pervasive games.

The design of the pervasive game experience involves gamers of all ages. Among these articles, pervasive games are designed for children mainly to exercise their reaction and thinking skills [50,51]. Subsequently, there are pervasive games designed for adults, teachers, and students to learn [32,44]. At the same time, there are pervasive games for older adults, indicating some positive effects on promoting physical activity [18]. From the literature, it can see some hints of the research tendencies of scholars: in the early days, they studied the pervasive game design experiences of children users, followed by the pervasive game design experiences of adult users, and in recent years, they focused on the pervasive game design experiences of older adult users. In particular, Santos, a Japanese scholar, and his research team have been studying the effects of social interaction mechanics in pervasive games on the physical activity levels of older adults in recent years [25]. They used multiple experimental measurements to thoroughly analyze older users’ perceptions of a pervasive game designed specifically for them, ranging from the game theme and visual style to game complexity and sociality. The direction of Santos’s research is very similar to the direction of this study, which is to investigate the effects of pervasive games on fitness among older adults, except that the players’ countries are different, the methods used are different, and the details of the specific participants studied are different. However, the research topics are very similar in terms of the effects of pervasive games on fitness among older adults. Nevertheless, the research conducted by Santos et al [25] is of great reference value to this study.

There are also some studies on service design for pervasive games, mainly from the evaluation perspective. This is probably because the service design concept is relatively new and has gradually emerged in design thinking and academic research in recent years. Perhaps it is therefore that the academic research perspective on pervasive games is still limited to simply evaluating the game itself. However, while evaluating the pervasive game itself, scholars are also exploring ways to improve and upgrade the pervasive game under study, which can be seen as an important manifestation of the service design concept. For example, refining the details of game mechanics and upgrading the details of game experience can be considered as research manifestations of service design for pervasive games. As in the case of El-Nasr et al [40] and Klaassen et al [28], the pervasive game for health programs was evaluated and measured for acceptability and integration into the participants’ lives, and at the same time, the pervasive game they studied was optimized and iterated. It is the best example of the service design philosophy running through the pervasive game. Of course, other studies also included such early concepts of service design.

### Limitations

The first limitation of this study is the database. This study only targeted 3 mainstream literature databases and did not include other databases, such as gray literature or PubMed. This may foreshadow the fact that, although the results obtained in this study through a systematic literature review method are authoritative, they could be more extensive and comprehensive. Another limitation of the study was that the search was conducted up to April 2022, and no articles were found in 2022 at the time of conducting the search, leaving an impression that the search left out publications of 2022.

### Future Studies

After a systematic analysis, it was found that none of the themes in this literature were related to branding design. However, the article by Kasapakis and Gavalas [36] on the pervasive game’s status, trends, and design principles can be considered a review of the existing successful pervasive games as branding. Pløhn et al [39] studied a dynamic and popular narrative approach to creating stories that can reinforce a sense of game maintenance and the brand of pervasive games. Valente et al [7] emphasized the need for nonfunctionality and highlighted aspects, such as cultural awareness, which can be seen as designing a brand strategy for pervasive games. Therefore, this literature does not explicitly go into the brand design of pervasive games but only touches on it to a greater or lesser extent. These results and findings were not coincident with the research object of brand design in this study. Thus, we believe that this systematic literature review has identified a research gap for branding design research on pervasive games and that it could be a future study direction.

### Conclusions

A new generation of applications that blend the real and digital worlds, such as pervasive games, presents daunting obstacles for software developers. This type of game has recently become a worldwide phenomenon, with thousands of people using their mobile phones to interact with the surrounding physical environment while walking down the street.

At the beginning of the *Introduction* section, an accurate description of the current situation of the game industry is provided, explaining the entertainment nature and economic value of the pervasive game. In the *Methods* section, this study adopted a rigorous systematic literature review research process with graphs and tables. This study identified 4 themes: design and development, interaction and technology, user and experience, and evaluation and service. Each of these 4 themes was subdivided into subsections and interpreted. Finally, in the *Discussion* section, the 4 themes are explained in terms of this study’s objectives: game design, interaction design, experience design, and service design. It also provides ideas for future research directions in pervasive games based on brand design.

## Abbreviations

AR: augmented reality
DPS: Dynamic Pervasive Storytelling
PRISMA: Preferred Reporting Items for Systematic Reviews and Meta-Analyses
SSR: smart substitute reality
VISOLE: Virtual Interactive Student-Oriented Learning Environment
